# Supplementary material for: Comparative multiomics analysis of cell physiological state after culture in a basket bioreactor
Source: Sci Rep. 2022 Nov 23;12:20161. doi: 10.1038/s41598-022-24687-4 (PMC9686226; doi:10.1038/s41598-022-24687-4)
Supplement: Supplementary file 1 — Supplementary Information 1. [file 41598_2022_24687_MOESM1_ESM.zip › raw data/Transcriptomic raw data/6.Enrichment/2.KEGG/TE1vsTC1/ALL/src/csab04360.html]

csab04360


- csab:103246295

- Up regulated genes

103246295(1.67927471583409)

- csab:103225530

- Up regulated genes

103225530(1.52855282236579)

- csab:103224900

- Down regulated genes

103224900(-1.42998371610984)

Close
